# Balance between Protection and Pathogenic Response to Aerosol Challenge with *Mycobacterium tuberculosis* (Mtb) in Mice Vaccinated with TriFu64, a Fusion Consisting of Three Mtb Antigens

**DOI:** 10.3390/vaccines9050519

**Published:** 2021-05-18

**Authors:** Sadaf Sulman, Benjamin O. Savidge, Kawther Alqaseer, Mrinal K. Das, Neda Nezam Abadi, John E. Pearl, Obolbek Turapov, Galina V. Mukamolova, M. Waheed Akhtar, Andrea May Cooper

**Affiliations:** 1 Department Respiratory Sciences, University of Leicester, Leicester LE1 7RH, UK; sadafsulman.sbs@pu.edu.pk (S.S.); bos8@le.ac.uk (B.O.S.); kawthera.hasan@uokufa.edu.iq (K.A.); mkd12@le.ac.uk (M.K.D.); nnezamabadi@ucc.ie (N.N.A.); jep38@le.ac.uk (J.E.P.); ot16@le.ac.uk (O.T.); gvm4@le.ac.uk (G.V.M.); 2 School of Biological Sciences, University of the Punjab, Lahore 54590, Pakistan; mwa.sbs@pu.edu.pk; 3 Leicester Tuberculosis Research Group—LTBRG, University of Leicester, Leicester LE1 7RH, UK; 4 Department of Basic Science, Faculty of Nursing, University of Kufa, P.O. Box 21, Kufa, Najaf Governorate, Najaf 540011, Iraq; 5 APC Microbiome Ireland, University College Cork, T12 YT20 Cork, Ireland

**Keywords:** tuberculosis, vaccine, antigen, weight loss

## Abstract

Tuberculosis vaccines capable of reducing disease worldwide have proven difficult to develop. BCG is effective in limiting childhood disease, but adult TB is still a major public health issue. Development of new vaccines requires identification of antigens that are both spatially and temporally available throughout infection, and immune responses to which reduce bacterial burden without increasing pathologic outcomes. Subunit vaccines containing antigen require adjuvants to drive appropriate long-lived responses. We generated a triple-antigen fusion containing the virulence-associated EsxN (Rv1793), the PPE42 (Rv2608), and the latency associated Rv2628 to investigate the balance between bacterial reduction and weight loss in an animal model of aerosol infection. We found that in both a low pattern recognition receptor (PRR) engaging adjuvant and a high PRR-engaging adjuvant (MPL/TDM/DDA) the triple-antigen fusion could reduce the bacterial burden, but also induced weight loss in the mice upon aerosol infection. The weight loss was associated with an imbalance between TNFα and IL-17 transcription in the lung upon challenge. These data indicate the need to assess both protective and pathogenic responses when investigating subunit vaccine activity.

## 1. Introduction

Tuberculosis (TB), caused by the intracellular pathogen *Mycobacterium tuberculosis* (Mtb), remains a leading cause of death from a single infectious agent and is estimated to kill approximately 1.2 million people every year [1]. Furthermore, up to a quarter of the world’s population are thought to be infected with Mtb with approximately 56 million of those at high risk of developing TB [2]. Vaccination is a key tool in the cost-effective control of infectious disease; however, development of a TB vaccine has proven difficult, although some progress is being made recently [3]. The choice of antigens in vaccine development is fundamental to efficient design. While there are practical considerations regarding how best to produce and deliver antigens, these are secondary to choosing specific antigens based on their ability to be detected by the immune system during infection. In TB, vaccines can be designed to stop infection, to prevent development of disease or to reduce the consequences of disease [4]. In the case of vaccines capable of stopping infection, antigen choice is straightforward but, due to the nature of Mtb infection [5], it is likely that vaccines that prevent disease rather than infection will be more easily achievable and, in this situation, antigen choice is critical. A key factor, therefore, that will define the efficacy of any one antigen is the temporal and spatial availability of the antigen throughout infection.

The widely used and cost-effective BCG (bacilli Calmette-Guerin) vaccine reduces disease development outside the lung but is only variably protective against lung disease [6]. We have postulated that, while acquired and memory immune responses are efficient at controlling disease outside the lung, their ability to control disease in the lung is influenced by the nature of the environment created by Mtb once deposited in the lung and prior to recognition by the acquired immune response [5,7,8,9]. It is this delay and the presence of a manipulated environment that makes the nature of the antigen important. Antigens produced by the bacterium have entirely unique spatial and temporal distributions depending upon their function during exponential growth, response to host defense mechanisms, role in modulating host cell function, and role in internal physiologic functions of the bacterium. These spatial and temporal distributions are then key to the ability of the acquired immune response to recognize them and mediate targeted functions within the Mtb-induced and manipulated inflammatory environment in the lung [5]. Indeed, vaccines such as ID93, designed with a view to combining antigens with different functions and temporal expression, are proving successful in trials [10,11].

In addition to defining the correct antigen, we need to define the correct immune response. While we have a high level of understanding of what constitutes the immune response to Mtb, we are not clear which elements of the immune response contribute to protection and which contribute to pathogenic outcomes. We have proposed that, while TB is predominantly a lung disease, it is by addressing the body as a whole that we will gain an understanding of the factors which promote active disease in those that are infected but in whom the disease is latent (latent TB infection—LTBI) [12]. While experimental medicine can provide insight into which clinical parameters are associated with poorer outcomes for the patient, we need to use animal models to investigate potential mechanistic mediators of both protection and pathology [13].

One key issue that limits our understanding is the fact that, while we can measure the presence of antigen-specific CD4 T cells, which are a key mediator of protection, in infected individuals, we cannot define how they perform at the site of disease or indeed whether they promote protection or pathogenic responses [5]. The ability of a T cell to mediate its function depends upon its antigen specificity, the conditions pertaining when it is both activated and when it is restimulated, and critically the availability of its cognate antigen at the site of infection. To investigate the role of these parameters, we have generated T cells to a variety of antigens by targeted vaccination, challenging these vaccinated animals, and then following not only the changes in bacterial burden but also the changes they mediate in the health of the animal.

Herein, we have selected antigens based on their immunogenicity in human populations and their association with different physiological activities of the bacterium. Specifically, we have generated a multi-epitope triple-antigen fusion, consisting of three Mtb antigens: the virulence associated EsxN (Rv1793), the PPE42 (Rv2608), and the latency associated Rv2628. EsxN is an ESAT-6 like early-stage antigen expressed by Mtb shortly after infection, which induces a strong T-cell immune response [14]. PPE42 drives strong humoral and variable T cell responses and has been shown to reduce bacterial burden in a mouse model when used as a vaccine [15]. Rv2628 is one of the eight DosR-regulated genes induced in response to stress and cytokine producing T-cells specific for this antigen are seen after prolonged infection [16]. These proteins have, therefore, varied temporal and spatial availability and we have combined these antigens into a triple-antigen fusion to determine the outcome in terms of bacterial growth and health consequences.

## 2. Materials and Methods

### 2.1. Generation and Production of Antigens

Recombinant triple-antigen fusion (TriFu64) was generated in *E. coli* C41 (DE3) containing pET15b::*TriFu64*. The plasmid was constructed by ligating DNA fragments encoding Mtb antigens Rv1793, Rv2628 and a truncated version of *Rv2608* (*tnPPE42*) into pET15b. The truncated version of Rv2608 was used due to its increased sensitivity in serologic studies, as reported previously for PstS1 [17]. The recombinant plasmids pET15b::*Rv1793*, pET15b::*Rv2628* and pET15b::*tnPPE42* (*tnRv2608*) were constructed as described previously [18]. Primers were designed to amplify genes of interest; restriction sites were introduced at the 5′ and 3′ ends of the primer sequences to facilitate cloning. The primer sequences are as follows: *Rv179*3-5′ GCC *CAT ATG* ACG ATT AAT TAC CAG TTC GGG; *Rv1793*-3′ ATA *AAG CTT* GGC CCA GCT GGA GCC GAC; *Rv2628*-5′ GAT *AAG CTT* ATG TCC ACG CAA CGA CCG; *Rv2628*-3′ TAC *GCT AGC* GAC CGC AAC GGC AAT CT; *tnPPE42*-5′ TTG *GCT AGC* GGG AAC CTG GGC AGC and *tnPPE42*-3′ ATA *GGA TCC* TTA GAA AAG TCG GGG TAG CGC. The DNA fragments were amplified from genomic DNA of Mtb H37Rv strain using Platinum Taq polymerase (ThermoFisher Scientific) with 34 cycles at 95 °C for 2 min, 95 °C for 30 sec, 55 °C for 30 sec, 72 °C for 1 min and 72 °C for 5 min. The gene products *Rv1793* and *Rv2628* were double digested with *NdeI/HindIII* and *HindIII/NheI* restriction enzymes, respectively, and cloned into pET15b-TEV vector. The plasmid was a double fusion gene construct *Rv1793-Rv2628*. Then the *tnPPE42* was digested with *NheI/BamHI* and ligated into the double fusion plasmid resulting in the TriFu64 plasmid consisting of the fusion gene construct *Rv1793-Rv2628-tnRv2608*, encoding a 64 kDa protein. The plasmid was sequenced by GATC Biotech (now Eurofins Genomics). The tnPPE42 and TriFu64 proteins were expressed in *E. coli* C41 (DE3) host cells and purified by immobilized metal affinity chromatography using Ni-NTA agarose, followed by gel filtration on CentriPure P100 (emp Biotech GmbH) column to remove imidazole. The purified proteins were analyzed using 4–20% SDS-PAGE precast gels (SERVA). Immunoblotting confirmed the presence of His-tagged proteins using monoclonal mouse poly-histidine antibody (1:3000 dilution) and anti-mouse antibody conjugated to alkaline phosphatase (1:10000 dilution) (both antibodies from Sigma-Aldrich, Haverhill, UK). Proteins from the pellet fraction were denatured and refolded using QuickFold™ Protein Refolding Kit (AthenaES, Baltimore, MD, USA) and they were purified using nickel chelating chromatography with HisPur^TM^ Ni-NTA resin. The level of bacterial LPS in the protein preparations was evaluated by *Limulus* amoebocyte lysate assay (ToxinSensor, GenScript USA Inc., Piscataway, NJ, USA). The ability of the triple-antigen fusion preparations to induce TNFα in murine macrophages, prepared as described in [19], was measured using an anti-TNFα ELISA kit (BioLegend).

An open-source program (http://www.protparam.net/index.html, accessed on 17 April 2018) was used to calculate the predicted molecular weight and extinction coefficient and thereby allow determination of the protein concentration using absorbance of the samples at 280 nm as measured by a Nanodrop spectrophotometer (ThermoFisher Scientific, Waltham, MA, USA). The protein samples were passed through 0.22 μm Millipore syringe filters and stored at −80 °C until use.

### 2.2. Animal Research Reporting of In Vivo Experiments [ARRIVE]

Study design: In vivo experimental design was based on a four-group study cohort in which the experimental unit was defined as a single mouse identified by ear punches. This four-group study design always included a sham control in which the animal received a vaccine lacking an antigen to provide a baseline response threshold. Sample size: The sample size was based on previously observed effect size for responses to ESAT-6 vaccination [20]. Inclusion and exclusion criteria: Animals which exhibited broken tissue around the vaccine site were excluded; all animals not specifically excluded were included in the study design. Randomization: Individual mice were randomly assigned to each of the four experimental groups and vaccinated accordingly. They were individually identifiable by ear-punches, avoiding potential bias in daily weighing or visual and behavioral health checks. Blinding: Since cages and mice were coded with respect to their experimental group assignment and weighing was performed by staff for whom the codes were unknowns, then the weights were determined in a blinded manner. Outcome measures: Several types of response measurements were derived from the interventions described, including the determination of bacterial load in organs, the in-vitro antigen-specific cytokine production response and the change in animal weight over the course of the experiments. In each of these outcome measures, bias was avoided through randomization and blinding. Animal statistical methods: Periodic animal weights were recorded by animal technicians using a digital scale accurate to hundredths of a gram. These weights were measured by placing a single unrestrained mouse in a low plastic container and waiting until the scale reported a stable reading. Between animals the low plastic container was cleaned and tared. Once the individual animal weights were unblinded, they were averaged by group, and a linear tread line was computed. Experimental animals: C57BL/6 were originally obtained from The Jackson Laboratory (Bar Harbor, Maine, US) through Charles River and bred in house at the University of Leicester under license. Both male and female mice between the ages of 6 to 12 weeks old were used at the time of the initial vaccination. Experimental interventions: Vaccination followed by infection.

### 2.3. Vaccination Protocols

The triple-antigen fusion and other antigens were delivered at a dose of approximately 0.5 µg per mouse suspended in either Imject Alum (ThermoFisher Scientific) according to the manufacturer’s protocol, or a combination of mono-phosphoryl lipid A (MPL) trehalose dicoryno-mycolate (TDM) and the small cationic molecule dimethyl dioctadecyl-ammonium bromide (DDA) or MPL/TDM/DDA as previously described [20]. ESAT6_1-20_ peptide was delivered in MPL/TDM/DDA formulated as described [20], but at 30 µg per mouse, this peptide vaccine was used as a positive control. Mice received the vaccination via a subcutaneous injection on the rump. Alum adjuvanted vaccinations were given three times with a 21-day period in between. MPL/TDM/DDA adjuvanted vaccines were delivered once. Mice were weighed before vaccination, weekly until day 15 then daily until the endpoint or day 30 post challenge. Mice were infected not less than 30 days post final vaccination.

### 2.4. Aerosol Infection and Bacterial Load Determination

The H37Rv strain of Mtb was grown in Proskauer Beck medium containing 0.05% Tween 80 to mid-log phase and frozen in 1 mL aliquots at −70 °C. For aerosol infections, subject animals were infected using a self-contained bespoke aerosol chamber (Walker Safety Cabinets Ltd., Glossop, UK) based on the ‘jet in air’ venturi nebulizer. A dose of approximately 100 colony forming units (CFU) was delivered. On day 1 and selected time points post-infection, infected mice were killed by anesthetic overdose and the organs were aseptically excised. Each of the organs was individually homogenized in saline using the Miltenyi Biotec gentleMACS Dissociator (Miltenyi Biotech, Bisley, UK), followed by plating serial dilutions of the organ homogenate on nutrient 7H11 agar (Sigma-Aldrich) c. Colony forming units were counted after 3 weeks of incubation at 37 °C [21].

### 2.5. Ethics Statement

All procedures involving live animals were carried out under UK Home Office license to AMC (P6DCE1A76) under an establishment license to the University of Leicester (X1798C4D2).

### 2.6. Lymphocyte Isolation for Antigen Stimulation

A single-cell suspension was prepared from the spleens of vaccinated mice by disrupting the organ using the Miltenyi Biotec gentleMACS Dissociator, followed by treatment with RBC lysis buffer. The cell suspension was then passed through a 70-µm nylon cell strainer, counted, and plated in U bottomed tissue culture plates at 2 × 10^5^ cells/mL. Cell suspensions were stimulated with peptides or protein preparations (5 ug/mL) and the supernatant analyzed using commercial ELISA test kits for TNFα and IL-17 (BioLegend, San Diego, CA, USA).

### 2.7. Real-Time PCR

RNA was extracted from total lung tissue homogenized in Trizol (Thermo Fisher Scientific). Trizol homogenates were processed using the RNeasy Kit (Qiagen GMBH, Hilden, Germany) as recommended by the manufacturer. RNA samples were treated with DNase (TURBO™ DNase, Thermo Fisher Scientific) and the cDNA amplification was carried out using RevertAid cDNA synthesis kit (Thermo Fisher Scientific). Amplified cDNA products were used or quantitative Real Time PCR (qRT-PCR) using SybrGreen as fluorescent probe (Fast SYBR™ Green Master Mix, Thermo Fisher Scientific). Fold increase in signal over that derived from sham vaccinated samples was determined using the ∆∆Ct calculation with normalization to b-actin transcription values. Oligonucleotide sequences were TNFα 5′-ACGGCATGGATCTCAAAGAC-3′, 5′-AGATAGCAAATCGGCTGACG-3′ [22]; IL-17 5′-TCAGCGTGTCCAAACACTGAG-3′, 5′-CGCCAAGGGAGTTAAAGACTT-3′ [23]; actin 5′-CTGGCTCCTAGCACCATGAAGAT-3′, 5′-GGTGGACAGTGAGGCCAGGAT-3′ [23].

### 2.8. Histology

The lower right lobe of each lung was inflated with 10% neutral buffered saline and processed routinely for light microscopy. Sections were then stained with hematoxylin and eosin. Slides were examined without knowledge of experimental group and subjectively graded for both quantity and quality of cellular accumulation as described previously [24,25].

### 2.9. Statistical Analysis

CFU data were Log_10_ transformed before analysis. GraphPad Prism Software was used to perform Student’s *t* tests or one-way ANOVA with appropriate multiple comparisons post-test (see figure legends). A *p* value ≤ 0.05 was considered significant.

## 3. Results

### 3.1. Truncated PPE42 (tnPPE42) and the 3-Antigen Fusion (TriFu64) Can Be Expressed and Purified

**Figure 1:**
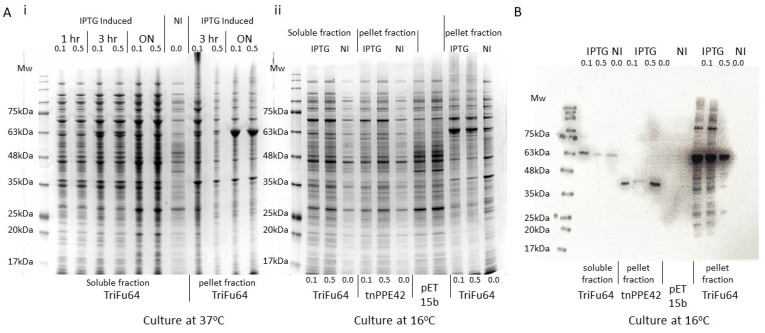
**Determination of conditions required to generate tnPPE42 and TriFu64 triple-antigen fusion.** Plasmids containing DNA fragments encoding the triple-antigen fusion genes were electroporated into *E. coli*, grown at 37 °C (**A**(**i**)) or 16 °C (**A**(**ii**),**B**) and induced with various concentrations of IPTG (0.0 (NI), 0.1 mM (0.1), or 0.5 mM (0.5)) for 1 h (1 hr), 3 h (3 hr) or overnight (ON). Both soluble and pellet fractions were generated, and the protein profile determined using 4–20% SDS-PAGE (**A**(**i**), **A**(**ii**)). (**B**) The presence of His-tagged proteins of the correct size in the soluble (sol. frac.) or pellet (pel. frac.) fraction was determined by Western blot analysis using an anti-poly histidine antibody.

**Figure 2:**
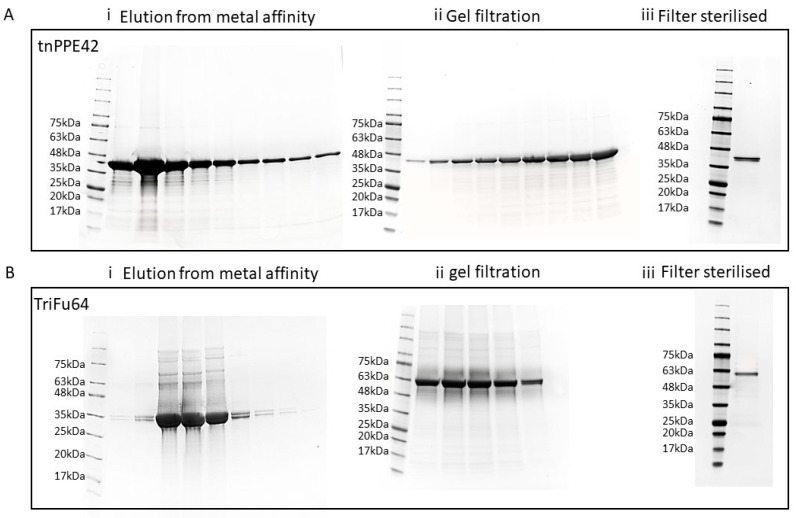
**Purification and sterilization of protein preparations.** (**A**) The soluble and pellet fractions from the induced *E. coli* cultures containing the tnPPE42 plasmid, and were filter sterilized ready for use in vivo. (**B**) The protein profile for each fraction is shown using 4–20% SDS-PAGE electrophoresis.

To investigate the ability of artificially combined triple-antigen fusion to influence the immune response to Mtb challenge, it was necessary to produce the proteins in amounts sufficient to deliver to mice and to ensure that the preparations did not result in non-specific activation of the immune response. To achieve this goal, we first identified proteins containing B and T cell epitopes which had been reported to be associated with protective responses in humans. We then created a recombinant triple-antigen fusion, TriFu64, composed of 3 Mtb antigens Rv1793, Rv2628, and truncated Rv2608 (tnPPE42). The DNA constructs encoding TriFu64 were inserted into the plasmids and expressed in *E.coli* under various inducing conditions. The SDS-PAGE separation of the cell lysates expressing tnPPE42 and TriFu64 constructs resulted in bands of the expected sizes, 41 kDa and 64 kDa, respectively (Figure 1A,B, whole gels and densitometry available [26]). By varying temperature, incubation time, and IPTG concentration, the production of protein was optimized, with both tnPPE42 and TriFu64 proteins being detected in the cell pellet in the form of inclusion bodies (Figure 1A,B). The presence of poly-histidine tagged proteins of the correct weight for both for both tnPPE42 and TriFu64 was confirmed (Figure 1B). The purified and sterilized protein preparations used for vaccination and restimulation of cultured cells are shown in Figure 2A (tnPPE42) and Figure 2B (TriFu64)(whole gels available [26]). The purified and sterilized preparations contained less than 25 endotoxin units per mg of protein and induced less than 23pg/mL of TNFα in primary macrophages (data available [26]). Together these data demonstrate that we were able to generate sufficient purified protein for vaccination and that the preparations were unlikely to induce strong non-specific immune responses.

### 3.2. TriFu64 Triple-Antigen Fusion Can Reduce Bacterial Burden in Aerosol Infected Mice

**Figure 3:**
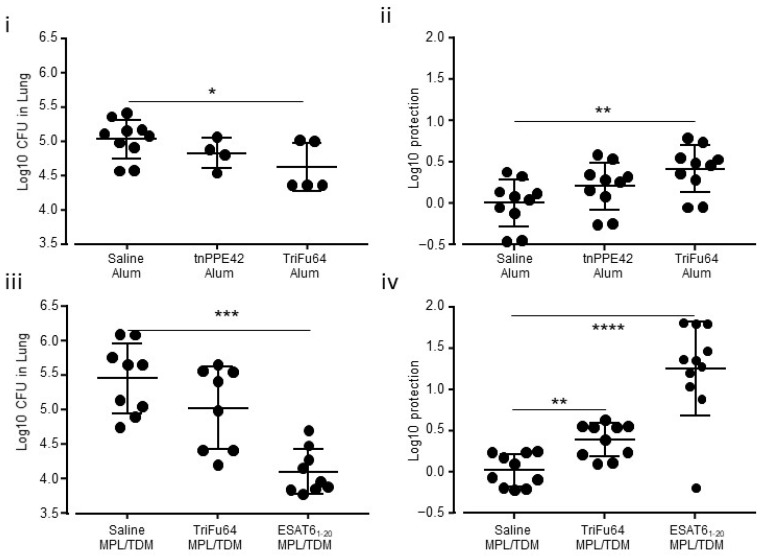
**Reduction in bacterial burden in vaccinated mice challenged with Mtb via the aerosol route.** Mice were vaccinated subcutaneously in the rump three times with protein antigens in alum (**i**,**ii**) or once with protein antigens in MPL/TDM/DDA (MPL/TDM)(**iii**,**iv**). Control mice received the adjuvant with saline (**i**–**iv**). Vaccinated mice were left to rest and then challenged no earlier than 30 days post vaccination via the aerosol route with approximately 100 colony forming units (CFU) of Mtb. Between 21 and 30 days later the mice were humanely killed, and the lungs homogenized and plated to determine the number of CFU in each lung (**i**,**iii**). The log10 protection is the difference between each value from the sham group and each of the experimental values for the vaccine formulation (**ii**,**iv**). Alum vaccine data is from 1 of 1 experiments *n* = 4–10 (**i**,**ii**). MPL/TDM/DDA (MPL/TDM) vaccine data (**iii**,**iv**) is from 2 of 2 experiments *n* = 6–9. Significance of differences between means was determined by ANOVA and Kruskal Wallis multiple comparison (* *p* ≤ 0.05, ** *p* ≤ 0.01, *** *p* ≤ 0.001, **** *p* ≤ 0.0001).

We hypothesized that the triple-antigen fusion would be able to induce protective immune responses against Mtb challenge when used as a vaccine. To test this hypothesis, we vaccinated mice with the TriFu64 or tnPPE42 in either alum or MPL/TDM/DDA. We chose to use alum to assess the ability of the triple-antigen fusion to work in the absence of strong co-delivery of pathogen associate molecular pattern (PAMP) as represented by the use of the MPL/TDM/DDA which works through a variety of pattern recognition receptors (PRR) to mediate its adjuvant activity. We used the aerosol delivery model to determine the impact of the TriFu64-based vaccine on the natural expression of immunity following low dose pathogen deposition in the lower airways. Using an alum adjuvant, we observed a modest reduction in bacterial burden in the lungs of TriFu64 vaccinated mice (Figure 3i,ii). The delivery of one antigen of the TriFu64 triple-antigen fusion, the truncated PPE42 (tnPPE42), did not, however, result in a significant reduction of the bacterial burden in the lungs (Figure 3i,ii).

We wanted to deliver TriFu64 in an adjuvant known to induce strong T cell responses which protect against Mtb challenge. To do this we delivered TriFu64 in MPL/TDM/DDA adjuvant and compared the response to a ESAT6_1-20_ peptide vaccine delivered in the same adjuvant, which we knew to induce protection in a reproducible manner [20]. We found that the TriFu64 triple-antigen fusion was able to induce approximately 0.5 log protection in the lung relative to the sham vaccinated mice (Figure 3iii, iv). Using the same adjuvant the ESAT6_1-20_ peptide vaccine was able to deliver 1 log of protection despite being at a lower concentration than used previously [20].

These data suggested that the TriFu64 triple-antigen fusion had some capacity to induce protection in a low dose aerosol challenge model both in the presence and absence of a PAMP-driven adjuvant.

### 3.3. The TriFu64 Triple-Antigen Fusion Induces a Strong Inflammatory Response upon Vaccination

**Figure 4:**
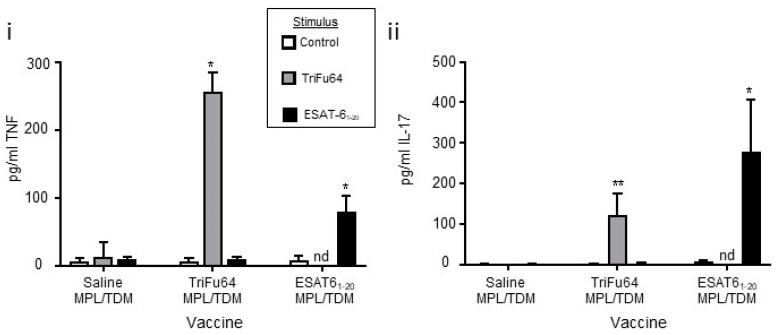
**Induction of differential antigen-specific cellular responses by MPL/TDM/DDA adjuvanted antigens.** Mice were vaccinated as described for Figure 3iii and then humanely killed 15 days post vaccination. Splenocytes from vaccinated mice were cultured either without stimulus (open bars) or with TriFu64 (light gray bars) or ESAT6_1-20_ (black bars). The supernatant was collected after 24 h of culture and analyzed for the cytokine’s TNFα (**i**) or IL-17 (**ii**) by ELISA. Data shows 1 experiment representative of 2 total with *n* = 4 for each. Significance of differences between means was determined by ANOVA and Kruskal Wallis comparison to control stimulus (* *p* ≤ 0.05*,* ** *p* ≤ 0.01).

Since vaccination with TriFu64 demonstrated modest protection in aerosol infection in both alum and MPL/TDM/DDA adjuvanted models, we wanted to determine the nature of the antigen-specific immune responses induced in the sham-, TriFu64- and ESAT6-vaccinated mice. To do this we restimulated splenocytes from the vaccinated mice with the TriFu64 or ESAT6_1-20_ antigens in vitro. Following this, we measured TNFα and IL-17 by ELISA in the supernatants. We did not detect reproducible signals from the splenocytes of the alum-vaccinated mice. We did, however, detect reproducible IL-17 and TNFα production by the splenocytes of MPL/TDM/DDA vaccinated mice (Figure 4).

We found that, when the TriFu64 triple-antigen fusion was used to restimulate splenocytes from sham-vaccinated mice, there was little TNFα induced. There was, however, a strong response in the TriFu64 vaccinated mice (Figure 4i dark gray bars), (TriFu64 was not used to restimulate ESAT6_1-20_ vaccinated mice). Restimulation of splenocytes from mice vaccinated with ESAT6_1-20_ peptide resulted in a TNFα response in the ESAT6_1-20_ vaccinated mice (Figure 4i black bars).

We found that, when the TriFu64 triple-antigen fusion was used to restimulate splenocytes from sham-vaccinated mice, there was no IL-17 produced. There was, however, a strong response in the TriFu64 triple-antigen fusion vaccinated mice (Figure 4ii dark gray bars). Restimulation of splenocytes from mice vaccinated with ESAT6_1-20_ peptide resulted in very little IL-17 response except in the ESAT6_1-20_ vaccinated mice (Figure 4ii black bars).

These data show that both the TriFu64 triple-antigen fusion and ESAT6_1-20_ drive both TNFα and IL-17 recall responses when adjuvanted with MPL/TDM/DDA.

### 3.4. The TriFu64 Triple-Antigen Fusion Impacts the Weight Gain in Mice Exposed to Aerosol Infection with Mtb

Protection against disease upon infection with Mtb is not simply a result of reduced bacterial burden; indeed, the immune response and vaccine-induced protection are not often associated with clearance of Mtb from the host. The ability to resist TB disease is a combination of a series of linked physiological parameters [12]. Before investigating ways in which to improve the TriFu64 triple-antigen fusion as a vaccine, we wanted to assess the impact of the vaccination on mouse health. To do this in a simple way, we weighed the mice weekly and then daily during the time when we know the immune response is expressed in the lungs of aerosol infected mice [20].

**Figure 5:**
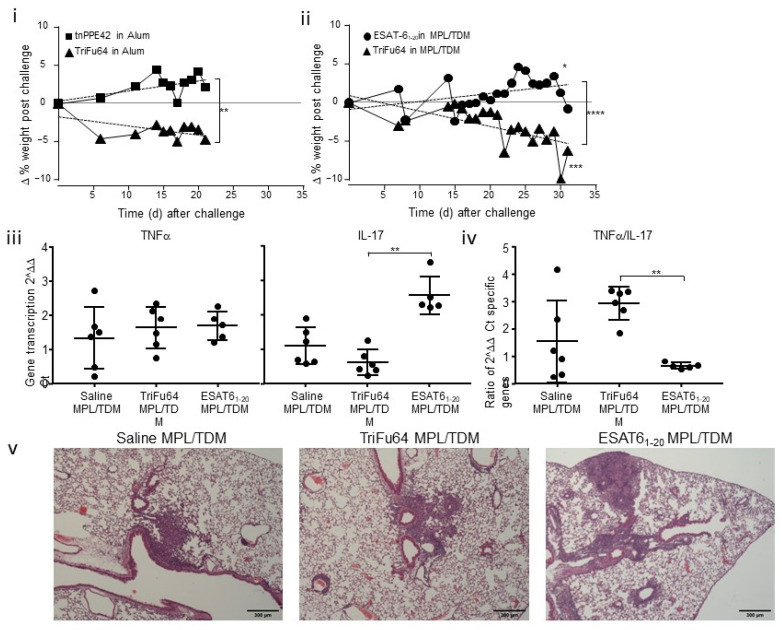
**Vaccination with TriFu64 results in weight loss during the response to aerosol infection.** Mice were vaccinated and then infected (as in Figure 3i,ii) and the weight of the mice relative to the weight on the day of challenge determined and baseline corrected against the sham values for each day. Vaccines used were tnPPE42 and TriFu64 in alum (**i**) and TriFu64 and ESAT6_1-20_ (**ii**–**v**) in MPL/TDM/DDA (MPL/TDM). Alum data (**i**) is 1 of 1 experiments n = 4–10. MPL/TDM/DDA (MPL/TDM) data (**ii**) is 2 experiments combined n = 8–9 and (**iii**–**v**) one experiment n = 5. Linear regression analysis was used to determine if lines were significantly different from each other, or if lines were significantly different from zero (* *p* ≤ 0.05, ** *p* ≤ 0.01, *** *p* ≤ 0.001, **** *p* ≤ 0.0001). Lung tissue was processed for transcriptional activity using RT-PCR and the ^2ΔΔCt value for TNFα and IL-17 determined (**iii**); the ratio of TNFα to IL-17 signal was determined within each sample (**iv**). Lung samples from each mouse were processed for histological analysis and representative 40X images are shown for each group (**v**). Significance of differences between means was determined by ANOVA and Kruskal Wallis comparison to the values from ESAT6_1-20_ MPL/TDM vaccinated and infected mice (** *p* ≤ 0.01).

We found that, even when delivered in a mild adjuvant, the TriFu64 triple-antigen fusion resulted in weight loss relative to the sham vaccinated mice (Figure 5i). In contrast, the tnPPE42 alum vaccinated mice did not differ significantly from the sham vaccinated mice in weight change (Figure 5i).

We undertook two further studies using the MPL/TDM/DDA vaccination (data have been combined in Figure 5ii). In one experiment the endpoint was 21 days and in the second it was 30 days. We found that delivery of TriFu64 in MPL/TDM/DDA resulted in significant weight loss during the immune response to Mtb challenge (Figure 5ii). In contrast to this weight loss, the ESAT6_1-20_ peptide delivered in MPL/TDM/DDA did not significantly reduce weight gain (Figure 5ii). Mice were monitored for general health (coat condition and activity) during the daily weighing and no other outward signs were noted.

To determine if the weight loss was associated with any altered immunological activity within the lung, we assessed gene transcription in the lungs of infected mice vaccinated with antigens in MPL/TDM/DDA adjuvant. We found that the infected sham-vaccinated mice exhibited variable TNFα expression and modest IL-17 expression. In contrast the infected ESAT6_1-20_ vaccinated mice had modest TNFα expression combined with a strong IL-17 response (Figure 5iii). When we determined the ratio of TNFα to IL-17 within each lung sample, it was clear that the sham vaccinated mice remained variable in their response. In contrast, the vaccinated mice exhibited a strongly polarized response with the ESAT6_1-20_ vaccinated mice having a 1:1 ratio of TNFα to IL-17, while the TriFu64 vaccinated mice had a three-fold higher level of TNFα to IL-17 in their lungs after challenge (Figure 5iv).

To investigate the impact of the vaccination on inflammatory outcomes, one lobe from each mouse was processed for histological analysis. We found that there were no major differences in the cellular components of the inflammatory infiltrate and there was more inflammation in both the TriFu64 and ESAT6_1-20_ vaccinated mice relative to the sham vaccinated mice (average score on five caudal lobes for Sham was 4, 8 for the TriFu64 and 12.8 for the ESAT6_1-20_ vaccinated mice).

These data indicate that vaccination with the TriFu64 results in weight loss upon Mtb challenge in both a low PRR-engaging adjuvant (alum) and a high PRR-engaging adjuvant such as MPL/TDM/DDA. There was no strong association with altered inflammatory cell accumulation and weight loss at the histological level. The data do, however, support an association between increased TNFα relative to IL-17 in the lungs of vaccinated, infected mice and weight loss during infection.

## 4. Discussion

Our study demonstrates that T cells induced by a single peptide in a high PRR-engaging adjuvant (MPL/TDM/DDA) can reduce bacterial burden without driving weight loss upon challenge. In contrast, a TriFu64 triple-antigen fusion delivered in both the high PRR-engaging and a low PRR-engaging adjuvant (alum) induces responses which can limit bacterial growth, but which also results in significant weight loss. Our studies show that, while vaccination with a triple-antigen fusion containing antigens with various temporal and spatial availability can reduce bacterial burden in the lung, there can be health consequences that need to be considered when balancing the potential impact of any vaccine.

Multiple-antigen fusions have been in development by several groups with a focus on antigenicity and breadth of functional activity [10]. ID93 in particular, a quadruple-antigen fusion containing four *Mtb* antigens Rv3619, Rv1813, Rv3620, and Rv2608, developed based on human responses to the antigens [11], is currently under clinical trial as a prophylactic, post-exposure, and a therapeutic vaccine [10] and is comprised of antigens from the actively secreted Esx family virulence factors, antigens associated with latency, and the PE/PPE antigen family [11]. We chose a similar grouping of antigens based on cellular and humoral human and animal responses associated with protection. While our data point to the potential for our triple-antigen fusion to have suboptimal outcomes, the current safety profile for ID93 is excellent [27]. It is clear, therefore, that while pathogenic outcomes may occur, it is the specific choice of antigens and model that define these outcomes rather than the simple fusion of the antigens or the combination of Esx, PPE, and latency associated antigens that is an issue.

We and others have previously shown that T cells vary in their ability to control bacterial growth and that this relates to their level of activation [28,29]. Others have also shown that the level of antigen availability also impacts the ability of the T cell to mediate protection [30,31]. It is therefore important to understand both how much and when the antigen is available following infection, when choosing antigen candidates. It is also important to understand how the activation of the T cell during vaccination impacts the ability of the memory T cell to respond to challenge. In this regard, comparison of adjuvants can lead to some insight. In our study, we compared the TriFu64 in both a low PRR mediated adjuvant and a high PRR engaging adjuvant and found that under both types of induction there was a reduction in weight upon challenge, that was not seen when the ESAT6_1-20_ peptide or the sham vaccination was used. When we consider one component protein of the TriFu64 triple-antigen fusion (tnPPE42), however, we found that in the low PRR engaging adjuvant it did not result in weight loss upon challenge. While this is a preliminary study, it is plausible that the potential pathological outcomes of a T cell response can be influenced not only by the antigen chosen but by the nature of the T cell activation during vaccination. This is not a surprising concept but supports the importance of T cell phenotyping following vaccination as a tool to identify pathogenic potential. With the increase in technology and T cell phenotyping capacity, this is a feasible approach to vaccine design.

Because we show that antigens can have both protective and pathogenic capacity when delivered in different adjuvants, our data support the need to pursue the use of subunit vaccines. These are particularly useful as they allow the formulation of vaccines that include only antigens with high protective and low pathogenic potential. The use of subunit vaccines as opposed to whole cell vaccines is appealing as it also removes the unknown variables that can be generated by variable culture conditions, such as has been seen for BCG generated over time and location [32]. We have used subunit vaccines in the past to define the function of memory T cell responses and the role of specific cytokines such as IL-17 in vaccine-induced protection [20]. We have also previously linked excessive IL-17 responses to pathologic consequences in the lung [33] and TNFα is a key regulator of inflammatory responses in mycobacterial disease [34]. Our study shows that both the TriFu64 and the ESAT6_1-20_ antigens delivered in MPL/TDM/DDA induce an antigen-specific IL-17 response but that the TriFu64 also induces a strong TNFα response. These differences could relate to the antigen dose which is a high single epitope for the peptide vaccine and a broad multi-epitope lower dose for the TriFu64. By combining T cell surface phenotype and cytokine-producing capacity we should be able to investigate the key elements of the vaccine that drive pathogenic potential in T cell induced responses using animal models, which may be useful in refining antigen and adjuvant choice in TB vaccine design.

What does the weight loss reflect in mice challenged with Mtb and undergoing an immune response modulated by vaccination? We show that vaccination with either ESAT6_1-20_ or TriFu64 induces TNFα and IL-17 antigen-specific responses with a tendency to high TNFα in the TriFu64 and high IL-17 in the ESAT6_1-20._ Upon infection, there is a very clear polarization in the TriFu64 vaccinated mice with a three-fold higher level of TNFα transcription than IL-17, while ESAT6_1-20_ vaccinated mice have equivalent transcriptional levels for these cytokines. At the current level of analysis, we did not see a strong association between the inflammatory cell accumulation and weight loss, with the most pronounced inflammatory activity being seen in the ESAT6_1-20_ vaccinated mice. The altered TNFα/IL-17 may result in reduced appetite and drinking activity. There are also physiologic/metabolic changes associated with TB [35]. While the short-term nature of our current model may not reflect these physiologic changes, any impact of vaccine-induced responses on parameters such as weight loss must be of concern in reviewing the ability of a vaccine to impact disease development in TB.

## 5. Conclusions

In conclusion, we show that combining Mtb antigens with various functional, spatial and temporal availability during infection we can induce inflammatory cytokines (IL-17 and TNFα) that can impact bacterial growth, but which also are also associated with weight loss upon aerosol infection. The implications of this work are that defining the function of specific T cells during vaccination and challenge is an important activity to undertake and that correlates of protection should also involve consideration of correlates of pathogenic potential.

## Data Availability

The data presented in this study are openly available in FigShare at [10.25392/leicester.data.14607879], reference number [26].
